# Preoperative Prediction of Meningioma Consistency *via* Machine Learning-Based Radiomics

**DOI:** 10.3389/fonc.2021.657288

**Published:** 2021-05-26

**Authors:** Yixuan Zhai, Dixiang Song, Fengdong Yang, Yiming Wang, Xin Jia, Shuxin Wei, Wenbin Mao, Yake Xue, Xinting Wei

**Affiliations:** Department of Neurosurgery, The First Affiliated Hospital of Zhengzhou University, Zhengzhou, China

**Keywords:** machine learning, consistency, meningioma, nomogram, radiomics

## Abstract

**Objectives:**

The aim of this study was to establish and validate a radiomics nomogram for predicting meningiomas consistency, which could facilitate individualized operation schemes-making.

**Methods:**

A total of 172 patients was enrolled in the study (train cohort: 120 cases, test cohort: 52 cases). Tumor consistency was classified as soft or firm according to Zada’s consistency grading system. Radiomics features were extracted from multiparametric MRI. Variance selection and LASSO regression were used for feature selection. Then, radiomics models were constructed by five classifiers, and the area under curve (AUC) was used to evaluate the performance of each classifiers. A radiomics nomogram was developed using the best classifier. The performance of this nomogram was assessed by AUC, calibration and discrimination.

**Results:**

A total of 3840 radiomics features were extracted from each patient, of which 3719 radiomics features were stable features. 28 features were selected to construct the radiomics nomogram. Logistic regression classifier had the highest prediction efficacy. Radiomics nomogram was constructed using logistic regression in the train cohort. The nomogram showed a good sensitivity and specificity with AUCs of 0.861 and 0.960 in train and test cohorts, respectively. Moreover, the calibration graph of the nomogram showed a favorable calibration in both train and test cohorts.

**Conclusions:**

The presented radiomics nomogram, as a non-invasive prediction tool, could predict meningiomas consistency preoperatively with favorable accuracy, and facilitated the determination of individualized operation schemes.

## Introduction

Meningioma is one of the most common intracranial tumors, with an incidence of 7.86 cases per 100,000 people per year (1). It can arise from any area where arachnoid cap cells are present. Current treatment options for meningioma include observation, surgery and radiosurgery (2). Though, radiosurgery may be a good choice for small tumor (<2 cm) (3), surgical resection is considered the primary treatment for patients with symptomatic meningiomas (4). However, the operative safe requires a thoroughly preoperative understanding of tumor characteristics and surgical anatomy.

The tumor consistency is one of the most important characteristics that affect surgical difficulty and degree of resection (5). Soft tumors can be removed by means of cutting and suctioning. However, firm tumors are more difficult to be removed, especially skull base meningiomas (6). More surgical instruments, such as ultrasonic aspiration, electrophysiological monitoring, and intraoperative navigation are needed. Thus, it is of vital importance to develop a noninvasive preoperative technique to predict tumor consistency. Previously, tumor consistency was predicted according to the signal intensity of T2 weighted images or Fluid attenuated inversion recovery images, but the accuracy was low (7). Radiomics has been considered as a potent approach for noninvasive high-throughput mining of tumor characteristics, which has been applied in many tumors, such as pituitary adenomas, gliomas (8, 9). Nonetheless, few studies have focused on radiomics signatures to predict meningioma consistency. Consequently, the current study aimed to establish a radiomics model for preoperative prediction of meningiomas’ consistency.

## Methods

### Patients

From January 2019 to May 2020, a total of 172 patients with meningiomas undergoing open craniotomy at the First Affiliated Hospital of Zhengzhou University were included in this study. The inclusion criteria were as follows: 1) meningioma diagnosis was confirmed by pathological report, 2) medical and imaging records were complete, 3) no history of medical treatment for meningioma. Excluding criteria were as follow: 1) incomplete medical records, 2) poor image quality, 3) preoperative treatment such as radiotherapy. The study was approved by the medical ethics committee of the First Affiliated Hospital of Zhengzhou University.

The patients were divided randomly into train cohort (n=120), which was used for model building, and test cohort (n=52), which was used for model validation. The following patients’ data were collected: clinical features (gender, age), conventional imaging features (tumor location, edema surrounding meningioma, CSF space surrounding meningioma), and pathology feature (WHO grade). The general characteristics of patients were displayed in **Table 1**. After surgery, the tumor consistency was classified as soft or firm according to Zada’s consistency grading system (10). Soft meningiomas were defined as those amenable to be removed totally or mainly with suction, which corresponding to Grade 1 and Grade 2 of Zada’s consistency grading system. Firm meningiomas were defined as those required sharp resection, ultrasonic aspiration or with calcified lesions, which corresponding to Grade 3, Grade 4 ad Grade 5 of Zada’s consistency grading system. We reviewed the surgical videos and operative recordings to determine the tumor consistency.

**Table 1:** General characteristics of patients.

	Train cohort (n=120)			Test cohort (n=52)		
	Soft	Firm	*P* value	Soft	Firm	*P* value
Age (mean, years)	52.8	52.4	0.86	53.3	55.5	0.62
Gender						
Male	7	18	0.90	2	10	0.75
Female	23	72		6	34	
Location						
Left	10	36	0.66	3	17	0.90
Right	15	44		3	19	
Midline	5	10		2	8	
Peritumoral edema						
No	22	57		5	27	
CSF space surrounding tumor						
Yes	17	51	1.0	5	29	0.83
No	13	39		3	15	
WHO grade						
WHO I	29	80	0.36	7	39	0.61
WHO II	1	10		1	5	

### MR Imaging Acquisition and Preprocessing

All patients underwent head MR imaging scan before surgery. Imaging was conducted on three models of MRI scanners, including Prisma, TrioTim and Verio (Siemens Healthineers, Erlangen, Germany). The MR imaging protocol included T1-weighted contrast-enhanced imaging (T1C), T2-weighted imaging (T2WI), Fluid attenuated inversion recovery imaging (FLAIR), Apparent diffusion coefficient imaging (ADC). The T1C sequence was acquired with the following range of parameters: repetition time (TR)/echo time (TE), 163-250/2.46-2.48msec; slice thickness, 5mm; spacing between slices, 6.50-6.75mm. The T2 sequence was acquired with the following range of parameters: TR/TE, 3900-5220/92-150msec; slice thickness, 5mm; spacing between slices, 6.50-6.75mm. The FLAIR sequence was acquired with the following range of parameters: TR/TE, 5000-8000/79-94msec; slice thickness, 5mm; spacing between slices, 6.50-6.75mm. The ADC sequence was acquired with the following range of parameters: TR/TE, 3000-4600/81-102msec; slice thickness, 5mm; spacing between slices, 6.50-6.75mm.

Preprocessing was performed in 3D-Slicer software (v4.9.0). First, image registration was performed to register T2WI, FLAIR, ADC sequence images to the T1C sequence images for each patient. Next, N4 bias field correction was applied to each sequence images to correct intensity non-uniformities.

### Tumor Segmentation and Feature Extraction

The region of interest (ROI) was manually drawn on T1C imaging by two neuroradiologists independently, using 3D-Slicer software. The neuroradiologists were blinded to the clinical data. The extraction of radiomic features was performed by using PyRadiomics package, which was an open-source python package for the extraction of radiomics features from medical imaging. The detail parameter settings of feature extraction were provided in the **Supplementary Document**. To avoid data heterogeneity bias, all MRI data were normalized (the intensity of image was scaled to 0-100) and resampled to the same resolution (3*3*3mm) before feature extraction. For each imaging sequence, three image types (original, Laplacian of Gaussian (LoG), wavelet) were applied, and six feature classes (shape, first order statistics, gray level cooccurrence matrix (glcm), gray level run length matrix (glrlm), gray level size zone matrix (glszm), gray level dependence matrix (gldm)) were calculated, which resulted in a total of 960 radiomic features (14 shape features, 18 first-order statistics features, 68 texture features, 172 LoG features, and 688 wavelet features). For each patient, a total of four imaging sequences were calculated, which generating 3840 radiomic features. Intraclass correlation coefficients (ICCs) analysis was performed on the two sets of ROIs, which were drawn by the two neuroradiologists. We defined the radiomic features with ICCs > 0.8 as stable features, which would be used in further analysis.

### Feature Selection and Establishment of Prediction Model

To avoid overfitting, feature selection was performed before model establishment. Features were selected by a two-stage process based on the radiomic features extracted by PyRadiomcs package. First, variances of each feature between soft and firm cases were calculated by t-test (11). Then, the features whose p-values of t-test were less than 0.05 were further analyzed by the least absolute shrinkage and selection operator (LASSO) regression algorithm. 10-fold cross-validation with a maximum area under the curve (AUC) criterion was performed to find the optimal λ. Finally, the features with non-zero coefficients were used to construct the prediction model, and the corresponding non-zero coefficients were defined as the Rad-score. The radiomics signature for each patient was generated using the linear combination of the values of selected features that were weighted by the Rad-score.

We applied five supervised machine-learning algorithms to establish the prediction model, including Random Forest (RF), K-nearest Neighbor (KNN), Support Vector Machine (SVM), Logistic Regression (LR), Adaboost Classifier (Ada), which generated 5 prediction models.

### Predictive Performance of Model

**Figure 1:**
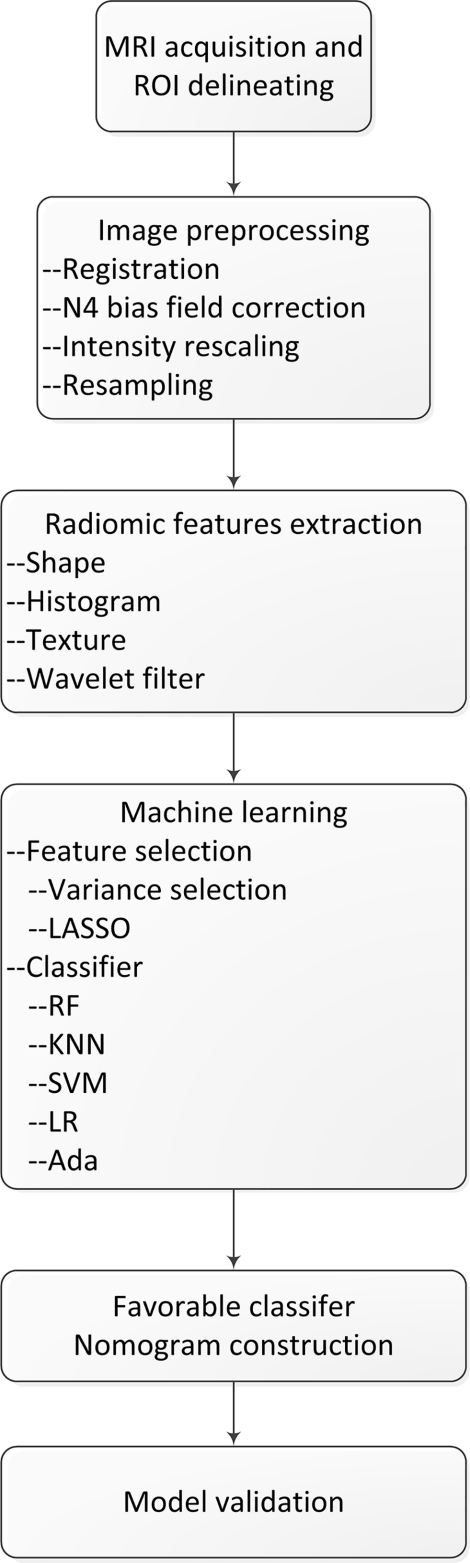
The flowchart of our study.

The test cohort was applied to evaluate performance of the model. The performance of both train and test cohorts was evaluated using AUC, sensitivity, specificity, and accuracy. The model with the highest AUC in test cohort was established as the final prediction model. The flowchart of this study is shown in **Figure 1**.

### Statistical Analysis

Differences in clinical characteristics between train and test cohort were assessed by Student’s t-test or chi-square test, as appropriate, and a two-sided p-value < 0.05 was considered statistically significant. Statistical analysis was conducted in Python (v3.7.6) and R software (v4.0.0).

## Results

### Patient Clinical Characteristics

A total of 172 patients were included in the study (120 cases in the train cohort, 52 cases in the test cohort). No significant differences between the soft and firm groups were detected in age, gender, tumor location, peritumoral edema, CSF space surrounding tumor and WHO grade.

### Feature Selection and Radiomic Machine-Learning Classifier Selection

In total, 3840 radiomics features were extracted in this study. 3719 radiomics features were stable features after being selected by ICCs. Finally, through variances selection and LASSO regression algorithm, 28 features were selected. The details of the selected features were shown in **Table 2**. The selected radiomics features were statistically different between the two tumor consistencies.

**Table 2:** The details of selected radiomics features.

Class	Feature name	Feature type	Sequence	Soft	Firm	*p*-value
Log filter (sigma=5.0mm)	glszm_GrayLevelNonUniformityNormalized	Texture	CET1	0.3152 ± 1.0513	-0.1098 ± 0.9469	0.0183
LLH wavelet filter	gldm_DependenceVariance	Wavelet	CET1	0.4287 ± 1.4918	-0.1169 ± 0.7826	0.0355
LHL wavelet filter	firstorder_Minimum	Wavelet	CET1	0.3146 ± 0.8763	-0.0993 ± 1.0174	0.0239
LHL wavelet filter	glszm_GrayLevelNonUniformityNormalized	Wavelet	CET1	0.2645 ± 1.0801	-0.1028 ± 0.9171	0.0379
LHH wavelet filter	glcm_MaximumProbability	Wavelet	CET1	0.3129 ± 1.1039	-0.0927 ± 0.9607	0.0277
HLL wavelet filter	firstorder_Entropy	Wavelet	CET1	-0.2845 ± 1.1074	0.0980 ± 0.9413	0.0351
HLL wavelet filter	firstorder_Uniformity	Wavelet	CET1	0.3099 ± 1.1197	-0.1033 ± 0.9389	0.0231
HLL wavelet filter	glszm_LargeAreaLowGrayLevelEmphasis	Wavelet	CET1	0.4034 ± 1.6223	-0.1508 ± 0.5664	0.045
HLH wavelet filter	firstorder_Mean	Wavelet	CET1	-0.3336 ± 1.0352	0.0725 ± 0.9484	0.0237
HHL wavelet filter	glrlm_ShortRunEmphasis	Wavelet	CET1	-0.3436 ± 1.2254	0.0965 ± 0.9164	0.045
HHL wavelet filter	gldm_DependenceVariance	Wavelet	CET1	0.4039 ± 1.5169	-0.1109 ± 0.7745	0.0498
HHH wavelet filter	firstorder_Maximum	Wavelet	CET1	-0.3615 ± 0.6751	0.1149 ± 1.0500	0.0012
Original	glszm_SmallAreaHighGrayLevelEmphasis	Texture	T2WI	0.2909 ± 1.2717	-0.0770 ± 0.9037	0.0459
Log filter (sigma=3.0mm)	firstorder_Mean	Histogram	T2WI	-0.5663 ± 0.9451	0.1659 ± 0.9639	0.0001
LHL wavelet filter	firstorder_Median	Wavelet	T2WI	-0.3615 ± 1.1908	0.0972 ± 0.9249	0.0125
HLL wavelet filter	firstorder_Median	Wavelet	T2WI	-0.3454 ± 1.0061	0.0841 ± 0.9758	0.0185
HLL wavelet filter	firstorder_Skewness	Wavelet	T2WI	0.3982 ± 1.0452	-0.1097 ± 0.9685	0.0056
HLL wavelet filter	glcm_Correlation	Wavelet	T2WI	0.3704 ± 1.0205	-0.1194 ± 0.9641	0.007
LLL wavelet filter	firstorder_10Percentile	Wavelet	T2WI	0.2768 ± 0.8975	-0.0912 ± 1.0122	0.0443
Original	glrlm_LongRunHighGrayLevelEmphasis	Texture	T2flair	0.3360 ± 1.1224	-0.0834 ± 0.9445	0.0218
Original	glszm_HighGrayLevelZoneEmphasis	Texture	T2flair	0.3152 ± 1.2024	-0.0777 ± 0.9196	0.0319
Log filter (sigma=3.0mm)	glcm_ClusterShade	Texture	T2flair	0.3666 ± 1.1785	-0.1018 ± 0.9300	0.0109
LLH wavelet filter	firstorder_Median	Wavelet	T2flair	-0.3452 ± 1.2631	0.1007 ± 0.9009	0.0475
HHH wavelet filter	firstorder_Mean	Wavelet	T2flair	0.2956 ± 0.8445	-0.0713 ± 1.0252	0.045
LHL wavelet filter	firstorder_Skewness	Wavelet	ADC	0.3221 ± 1.0248	-0.0928 ± 0.9849	0.0244
HLL wavelet filter	firstorder_Skewness	Wavelet	ADC	0.3258 ± 1.0802	-0.1050 ± 0.9557	0.0183
HLH wavelet filter	firstorder_Median	Wavelet	ADC	0.2946 ± 0.7405	-0.1275 ± 0.9200	0.0102
HLH wavelet filter	firstorder_Skewness	Wavelet	ADC	-0.2725 ± 1.1742	0.0911 ± 0.9284	0.0467

The performances of the five prediction models were shown in **Table 3**. Notably, the LR and Ada models performed well in the validation, with AUCs of 0.83 and 0.82, respectively. The sensitivities of LR and Ada models were 0.91 and 0.89 in the test cohort, respectively, and the accuracies were 0.88 and 0.87 in the test cohort, respectively, the F1-scores were 0.93 and 0.92 in the test cohort, respectively. It revealed that LR model performed best.

**Table 3:** The performances of five prediction models.

Comparisons	Cohorts	RF	KNN	SVM	LR	Ada
AUC	Train	1.0	0.95	1.0	0.89	1.0
	Test	0.56	0.67	0.73	0.83	0.82
Sensitivity	Train	1.0	0.91	1.0	0.87	1.0
	Test	1.0	0.84	0.95	0.91	0.89
Specificity	Train	1.0	0.99	1.0	0.92	1.0
	Test	0.13	0.50	0.50	0.75	0.75
Accuracy	Train	1.0	0.95	1.0	0.89	1.0
	Test	0.87	0.79	0.88	0.88	0.87
F1-score	Train	1.0	0.95	1.0	0.89	1.0
	Test	0.93	0.87	0.93	0.93	0.92

RF, Random Forest; KNN, K-nearest Neighbor; SVM, Support Vector Machine; LR, Logistic Regression; Ada, Adaboost Classifier; AUC, Area Under the Curve.

### Evaluation of Radiomics Signature and Clinical Risk Factors

**Figure 2:**
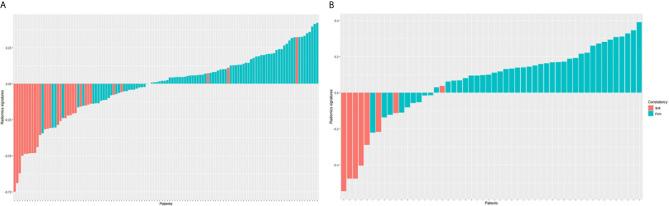
Radiomics signature for each patient in the train cohort **(A)** and test cohort **(B)**. The red bars show the radiomics signature values for the soft meningiomas, and the blue bars show the values for the firm meningiomas.

The radiomics signatures for each patient in train and test cohorts were calculated. The formula of radiomics signatures was presented in **Supplementary Document**. The soft tumors presented lower radiomics signatures than firm tumors (see **Figure 2**). The mean radiomics signature of soft tumor in train cohort was -0.286, which was significantly lower than that of firm tumor (0.075, *p*<0.001). In test cohort, the mean radiomics signatures of soft and firm tumors were -0.311 and 0.122, respectively (*p*<0.001). Radiomics signature and clinical factors were further analyzed using logistic regression to identified the independent predictors of meningioma consistency. The univariate logistic regression showed that only radiomics signature was the significant prediction factor. The logistic regression results were listed in **Table 4**. It showed that only radiomics signature was the independent predictor.

**Table 4:** The logistic regression results of radiomics signature and clinical risk factors.

	Univariate logistic regression	
	OR (95%CI)	*P* value
Gender (female vs male)	1.074 (0.712-1.367)	0.851
Age	0.987 (0.960-1.014)	0.343
Peritumoral edema (yes vs no)	0.954 (0.520-1.747)	0.877
Tumor location (right side or middle vs left side)	0.947 (0.599-1.496)	0.816
CSF space surrounding tumor (yes vs no)	1.094 (0.607-1.974)	0.764
Radiomics signature	1407.372 (202.969-13879.683)	<0.001

### Radiomics Nomogram Construction and Validation

**Figure 3:**
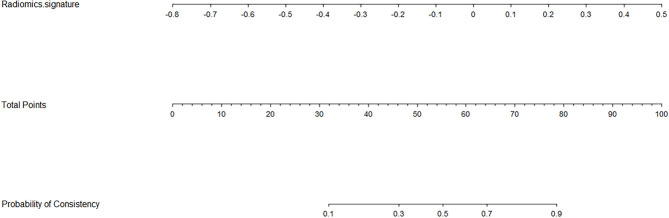
Radiomics nomogram for the meningiomas consistency. As an example, if one patient had the radiomics signature of -0.2, the corresponding total points was about 46, which corresponding to a 30% probability of a firm meningioma. That’s to say, using the nomogram, the patient’s meningioma consistency was predicted to be soft before surgery.

**Figure 4:**
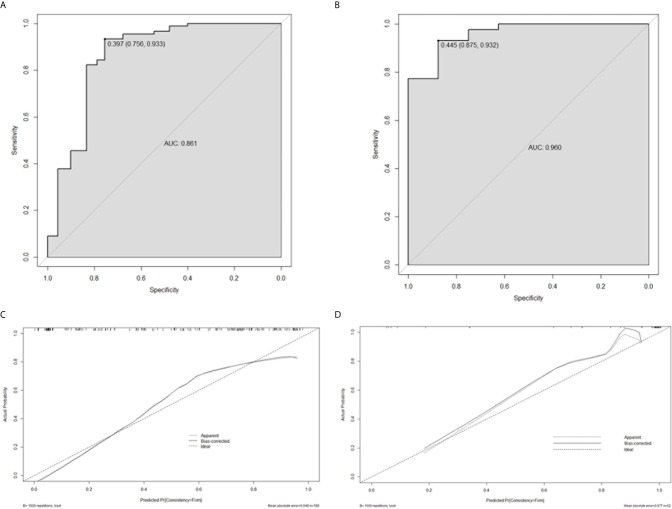
The performance evaluation of the radiomics nomogram. **(A)** the ROC curve in train cohort; **(B)** the ROC curve in test cohort; **(C)** the calibration curve in train cohort; **(D)** the calibration curve in test cohort.

Based on the logistic regression, a radiomics nomogram was constructed to make it easier to use clinically (see **Figure 3**). According to the radiomics signature, the probability of firm meningioma was obtained. The ROC curve was used to evaluate the sensitivity and specificity of the nomogram (see **Figure 4**). The nomogram showed a good sensitivity and specificity with AUCs of 0.861 and 0.960 in train and test cohorts, respectively. Moreover, the calibration graph of the nomogram showed a favorable calibration in both train and test cohorts (see **Figure 4**). These findings revealed the satisfying ability of the radiomics nomogram to classify meningiomas consistency.

**Figure 5:**
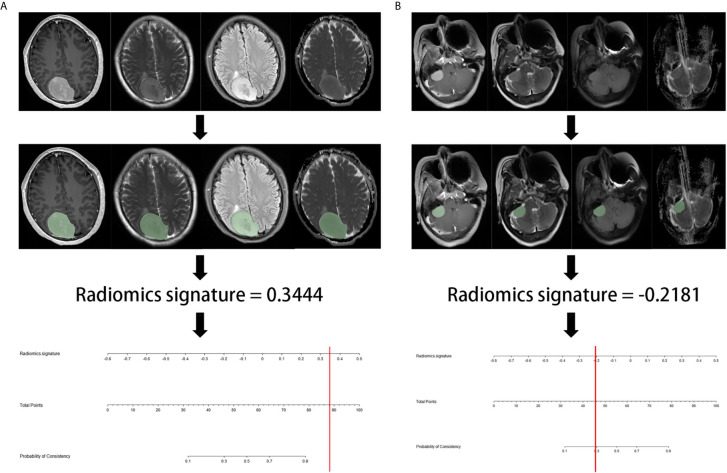
The example flowchart of prediction. **(A)** after ROI delineating, image preprocessing, the value of radiomics signature was 0.3444, which was calculated by the python script including radiomics extraction and model calculation. The result corresponded to >90% probability of a firm consistency. Thus, the meningioma consistency was predicted to be firm, which was confirmed in surgery. **(B)** the radiomics signature was -0.2181, which corresponding to a 30% probability of a firm consistency. Thus, the meningioma consistency was predicted to be soft, which was confirmed in7nbsp;surgery.

**Figure 5** showed the flowchart of prediction. We wrote a python script to facilitate radiomics signature calculation, which was provided in the **Supplementary Document**.

## Discussion

Meningiomas are intracranial extra-axial lesion, which are primarily managed by operation. About 40% of meningiomas patients can achieve Simpson I resection, while 35% achieve Simpson II (12). It has been reported that the risk factors of incomplete resection are skull-base location, bone invasion, firm consistency, adhesion to vessels (13). Multiple studies have reported the significance of meningiomas’ consistency to determine surgical planning and length of operation time. Especially for meningiomas in skull base area, firm tumor may need more instruments, such as ultrasonic aspirator. Therefore, determination of meningiomas consistency before surgery is important to make the operation plan, avoid the multistage surgical procedure.

There have been several studies that make efforts to predict the consistency of meningiomas. Most of the literatures predict tumor consistency utilizing the conventional MRI techniques. Many studies have reported that hyperintensity on T2WI was associated with soft consistency (14). However, Kashimura et al. reported that there was no association between T2WI intensity and consistency (15). Romani et al. also reported negative results using T1WI, T2WI or FLAIR sequences (16). Both of sensitivity and specificity were low using the conventional MRI prediction method, which providing limited information of consistency before the operation.

Radiomics is a new area of study in which quantitative and high-throughput data are extracted, processed and analyzed to explore their relationships with valuable information. Radiomics technique and machine learning algorithm have been widely used in many tumors’ differential diagnosis and consistency prediction before operation (17–20). Yang Zhang et al. developed a radiomics model that could be used in discrimination of lesions located in the anterior skull base (8). In glioblastoma, Xi Zhang reported a radiomics nomogram including 25 selected features, which performing better than clinical risk factors in survival stratification, and the C-index reached up to 0.974 (21).

Only one article that using radiomics features to predict meningiomas consistency was published (22). The author established a model with the Naive Bayes algorithm with an AUC of 0.961. However, the enrolled cases were few, and the model was not validated in the test group, which reduced the reliability. In our study, we have certain advantages. Firstly, a total of 172 patients were enrolled. The large sample size provided reliable results. Secondly, the patients were divided into train and test cohorts. The prediction model was validated in test group for internal validation. The result showed that the AUC in test cohorts was up to 0.960, which meaning that the constructed model can successfully classify soft and firm meningiomas. Thirdly, the model displayed good calibration and discrimination. Fourth, we provided a python script, which could calculate the radiomics signature conveniently. With the help of radiomics nomogram, neurosurgeon can get the consistency prediction result accurately.

This study also had some limitations. First, although we had validated the model in the test cohort, this was not a multicenter study. More prospective datasets are needed for independent verification of the robustness and repeatability of the radiomics nomogram. Second, the patients’ MRI imaging were acquired by different scanners, which would increase the data heterogeneity bias. To avoid it, all MRI imaging were subjected to imaging normalization before feature extraction. Finally, although variance selection and LASSO regression methods were highly efficient, they may be less stable when huge number of features were involved in the model. Other feature selection methods should be investigated in the future work.

In conclusion, our study developed and validated a radiomics nomogram based on the multiparametric MRI imaging. The radiomics nomogram demonstrated a favorable predictive accuracy of meningiomas consistency before surgery, which showing the potential of clinical application.

## Data Availability Statement

The raw data supporting the conclusions of this article will be made available by the authors, without undue reservation.

## Ethics Statement

The studies involving human participants were reviewed and approved by Ethics Committee of First Affiliated Hospital of Zhengzhou University. The patients/participants provided their written informed consent to participate in this study.

## Author Contributions

YZ and XW designed the study. YW, XJ, SW and WM collected clinical and radiomics data. DS, FY and YX pre-processed patients MR imaging and drew the ROI. YZ and DS analyzed the data and developed prediction model. YZ wrote the manuscript. All authors contributed to the article and approved the submitted version.

## Funding

Joint Construction Project of Medical Science and Technology Planning Project of Henan Province. Grant No.LHGJ20190104.

## Conflict of Interest

The authors declare that the research was conducted in the absence of any commercial or financial relationships that could be construed as a potential conflict of interest.

## References

[1] Preusser M Brastianos PK Mawrin C. Advances in Meningioma Genetics: Novel Therapeutic Opportunities. Nat Rev Neurol (2018) 14:106–15. 10.1038/nrneurol.2017.168 29302064

[2] Apra C Peyre M Kalamarides M. Current Treatment Options for Meningioma. Expert Rev Neurother (2018) 18:241–9. 10.1080/14737175.2018.1429920 29338455

[3] Gupta A Xu Z Cohen-Inbar O Snyder MH Hobbs LK Li C. Treatment of Asymptomatic Meningioma With Gamma Knife Radiosurgery: Long-Term Follow-Up With Volumetric Assessment and Clinical Outcome. Neurosurgery (2019) 85:E889–99. 10.1093/neuros/nyz126 31062018

[4] Zeng L Wang L Ye F Chen J Lei T Chen J. Clinical Characteristics of Patients With Asymptomatic Intracranial Meningiomas and Results of Their Surgical Management. Neurosurg Rev (2015) 38:481–88; discussion 488. 10.1007/s10143-015-0619-1 25697143

[5] Itamura K Chang KE Lucas J Donoho DA Giannotta S Zada G. Prospective Clinical Validation of a Meningioma Consistency Grading Scheme: Association With Surgical Outcomes and Extent of Tumor Resection. J Neurosurg (2018) 131(5):1347–682. 10.3171/2018.7.JNS1838 30554187

[6] Little KM Friedman AH Sampson JH Wanibuchi M Fukushima T. Surgical Management of Petroclival Meningiomas: Defining Resection Goals Based on Risk of Neurological Morbidity and Tumor Recurrence Rates in 137 Patients. Neurosurgery (2005) 56:546–59; discussion 546-559. 10.1227/01.NEU.0000153906.12640.62 15730581

[7] Karthigeyan M Dhandapani S Salunke P Singh P Radotra BD Gupta SK. The Predictive Value of Conventional Magnetic Resonance Imaging Sequences on Operative Findings and Histopathology of Intracranial Meningiomas: A Prospective Study. Neurol India (2019) 67:1439–45. 10.4103/0028-3886.273632 31857531

[8] Zhang Y Shang L Chen C Ma X Ou X Wang J. Machine-Learning Classifiers in Discrimination of Lesions Located in the Anterior Skull Base. Front Oncol (2020) 10:752. 10.3389/fonc.2020.00752 32547944 PMC7270197

[9] Wang J Zheng X Zhang J Xue H Wang L Jing R. An MRI-based Radiomics Signature as a Pretreatment Noninvasive Predictor of Overall Survival and Chemotherapeutic Benefits in Lower-Grade Gliomas. Eur Radiol (2021) 31(4):1785–94. 10.1007/s00330-020-07581-3 33409797

[10] Zada G Yashar P Robison A Winer J Khalessi A Mack WJ. A Proposed Grading System for Standardizing Tumor Consistency of Intracranial Meningiomas. Neurosurg Focus (2013) 35:E1. 10.3171/2013.8.FOCUS13274 24289117

[11] Parmar C Grossmann P Bussink J Lambin P Aerts H. Machine Learning Methods for Quantitative Radiomic Biomarkers. Sci Rep (2015) 5:13087. 10.3389/fonc.2015.00272 26278466 PMC4538374

[12] Lemee JM Corniola MV Da Broi M Joswig H Scheie D Schaller K. Extent of Resection in Meningioma: Predictive Factors and Clinical Implications. Sci Rep (2019) 9:5944. 10.1038/s41598-019-42451-z 30976047 PMC6459829

[13] Shiroishi MS Cen SY Tamrazi B D’Amore F Lerner A King KS. Predicting Meningioma Consistency on Preoperative Neuroimaging Studies. Neurosurg Clin N Am (2016) 27:145–54. 10.1016/j.nec.2015.11.007 PMC4936899 27012379

[14] Watanabe K Kakeda S Yamamoto J Ide S Ohnari N Nishizawa S. Prediction of Hard Meningiomas: Quantitative Evaluation Based on the Magnetic Resonance Signal Intensity. Acta Radiol (2016) 57:333–40. 10.1177/0284185115578323 25824207

[15] Kashimura H Inoue T Ogasawara K Arai H Otawara Y Kanbara Y. Prediction of Meningioma Consistency Using Fractional Anisotropy Value Measured by Magnetic Resonance Imaging. J Neurosurg (2007) 107:784–7. 10.3171/JNS-07/10/0784 17937223

[16] Romani R Tang WJ Mao Y Wang DJ Tang HL Zhu FP. Diffusion Tensor Magnetic Resonance Imaging for Predicting the Consistency of Intracranial Meningiomas. Acta Neurochir (Wien) (2014) 156:1837–45. 10.1007/s00701-014-2149-y 25002281

[17] Lao J Chen Y Li ZC Li Q Zhang J Liu J. A Deep Learning-Based Radiomics Model for Prediction of Survival in Glioblastoma Multiforme. Sci Rep (2017) 7:10353. 10.1038/s41598-017-10649-8 28871110 PMC5583361

[18] Han W Qin L Bay C Chen X Yu KH Miskin N. Deep Transfer Learning and Radiomics Feature Prediction of Survival of Patients With High-Grade Gliomas. AJNR Am J Neuroradiol (2020) 41:40–8. 10.3174/ajnr.A6365 PMC6975328 31857325

[19] Fan Y Hua M Mou A Wu M Liu X Bao X. Preoperative Noninvasive Radiomics Approach Predicts Tumor Consistency in Patients With Acromegaly: Development and Multicenter Prospective Validation. Front Endocrinol (Lausanne) (2019) 10:403. 10.3389/fendo.2019.00403 31316464 PMC6611436

[20] Li L Wang K Ma X Liu Z Wang S Du J. Radiomic Analysis of Multiparametric Magnetic Resonance Imaging for Differentiating Skull Base Chordoma and Chondrosarcoma. Eur J Radiol (2019) 118:81–7. 10.1016/j.ejrad.2019.07.006 31439263

[21] Zhang X Lu H Tian Q Feng N Yin L Xu X. A Radiomics Nomogram Based on Multiparametric MRI Might Stratify Glioblastoma Patients According to Survival. Eur Radiol (2019) 29:5528–38. 10.1007/s00330-019-06069-z 30847586

[22] Cepeda S Arrese I García-García S Velasco-Casares M Escudero-Caro T Zamora T. Meningioma Consistency can Be Defined by Combining the Radiomic Features of Magnetic Resonance Imaging and Ultrasound Elastography. A Pilot Study Using Machine Learning Classifiers. World Neurosurg (2020) 146:e1147–59. 10.1016/j.wneu.2020.11.113 33259973

