# Supplementary material for: Preoperative Prediction of Meningioma Consistency via Machine Learning-Based Radiomics
Source: Front Oncol. 2021 May 26;11:657288. doi: 10.3389/fonc.2021.657288 (PMC8187861; doi:10.3389/fonc.2021.657288)
Supplement: Supplementary file 1 [file DataSheet_1.docx]

**Data Supplement S1: detail parameter settings of feature extraction in PyRadiomics package.**

imageType:

Original: {}

LoG:

sigma: [3.0, 5.0]

Wavelet: {}

featureClass:

shape:

firstorder:

glcm:

- 'Autocorrelation'

- 'JointAverage'

- 'ClusterProminence'

- 'ClusterShade'

- 'ClusterTendency'

- 'Contrast'

- 'Correlation'

- 'DifferenceAverage'

- 'DifferenceEntropy'

- 'DifferenceVariance'

- 'JointEnergy'

- 'JointEntropy'

- 'Imc1'

- 'Imc2'

- 'Idm'

- 'Idmn'

- 'Id'

- 'Idn'

- 'InverseVariance'

- 'MaximumProbability'

- 'SumEntropy'

- 'SumSquares'

glrlm:

glszm:

gldm:

setting:

normalize: true #false in ADC maps

normalizeScale: 100

interpolator: 'sitkBSpline'

resampledPixelSpacing: [3, 3, 3]

binWidth: 5

voxelArrayShift: 300

label: 1

**Data Supplement S2: Radiomics signature calculation formula**

Radiomics signature=

(-0.0879) * ['T1c_log-sigma-5-0-mm-3D_glszm_GrayLevelNonUniformityNormalized']

+(-0.0198) * ['T1c_wavelet-LLH_gldm_DependenceVariance']

+(-0.0347) * ['T1c_wavelet-LHL_firstorder_Minimum']

+(-0.0048) * ['T1c_wavelet-LHL_glszm_GrayLevelNonUniformityNormalized']

+(0.0113) * ['T1c_wavelet-LHH_glcm_MaximumProbability']

+(-0.0363) * ['T1c_wavelet-HLL_firstorder_Entropy']

+(0.0031) * ['T1c_wavelet-HLL_firstorder_Uniformity']

+(-0.0470) * ['T1c_wavelet-HLL_glszm_LargeAreaLowGrayLevelEmphasis']

+(0.0636) * ['T1c_wavelet-HLH_firstorder_Mean']

+(0.0010) * ['T1c_wavelet-HHL_glrlm_ShortRunEmphasis']

+(-0.0279) * ['T1c_wavelet-HHL_gldm_DependenceVariance']

+(0.0211) * ['T1c_wavelet-HHH_firstorder_Maximum']

+(-0.0461) * ['T2_original_glszm_SmallAreaHighGrayLevelEmphasis']

+(0.0721) * ['T2_log-sigma-3-0-mm-3D_firstorder_Mean']

+(0.0143) * ['T2_wavelet-LHL_firstorder_Median']

+(0.0026) * ['T2_wavelet-HLL_firstorder_Median']

+(-0.0288) * ['T2_wavelet-HLL_firstorder_Skewness']

+(-0.0650) * ['T2_wavelet-HLL_glcm_Correlation']

+(-0.0293) * ['T2_wavelet-LLL_firstorder_10Percentile']

+(-0.0019) * ['T2flair_original_glrlm_LongRunHighGrayLevelEmphasis']

+(-0.0192) * ['T2flair_original_glszm_HighGrayLevelZoneEmphasis']

+(-0.0517) * ['T2flair_log-sigma-3-0-mm-3D_glcm_ClusterShade']

+(0.0181) * ['T2flair_wavelet-LLH_firstorder_Median']

+(-0.0179) * ['T2flair_wavelet-HHH_firstorder_Mean']

+(-0.0437) * ['ADC_wavelet-LHL_firstorder_Skewness']

+(-0.0140) * ['ADC_wavelet-HLL_firstorder_Skewness']

+(-0.0983) * ['ADC_wavelet-HLH_firstorder_Median']

+(0.0355) * ['ADC_wavelet-HLH_firstorder_Skewness']

**Data Supplement S3: the python script of radiomics signature calculation**

**# -*- coding: utf-8 -*-**

**import tkinter as tk**

**import radiomics.featureextractor as FEE**

**def T1c():**

**ori_path=entry1.get()**

**lab_path=entry5.get()**

**para_path='param.yaml'**

**extractor= FEE.RadiomicsFeatureExtractor(para_path)**

**result= extractor.execute(ori_path, lab_path)**

**T1C_value = ((-0.0879) * (result['log-sigma-5-0-mm-3D_glszm_GrayLevelNonUniformityNormalized']-0.0496722822023121)/0.016580929209522**

**+(-0.0198) * (result['wavelet-LLH_gldm_DependenceVariance']-2.80000997710982)/2.48108709989194**

**+(-0.0347) * (result['wavelet-LHL_firstorder_Minimum']+230.038254390231)/100.059373429889**

**+(-0.0048) * (result['wavelet-LHL_glszm_GrayLevelNonUniformityNormalized']-0.0280935086820809)/0.0083008353540124**

**+(0.0113) * (result['wavelet-LHH_glcm_MaximumProbability']-0.0639668250057803)/0.0347979260652301**

**+(-0.0363) * (result['wavelet-HLL_firstorder_Entropy']-5.31659457573988)/0.496598183048982**

**+(0.0031) * (result['wavelet-HLL_firstorder_Uniformity']-0.0345500586589595)/0.0127316075058006**

**+(-0.0470) * (result['wavelet-HLL_glszm_LargeAreaLowGrayLevelEmphasis']-0.0180893810693641)/0.0302961747308273**

**+(0.0636) * (result['wavelet-HLH_firstorder_Mean']-0.0637406382138728)/1.05561873005995**

**+(0.0010) * (result['wavelet-HHL_glrlm_ShortRunEmphasis']-0.967566985641618)/0.0154068102509531**

**+(-0.0279) * (result['wavelet-HHL_gldm_DependenceVariance']-1.92187294864161)/1.69262435797558**

**+(0.0211) * (result['wavelet-HHH_firstorder_Maximum']-50.315030553971)/23.9348605640564)**

**return T1C_value**

**def T2():**

**ori_path=entry2.get()**

**lab_path=entry5.get()**

**para_path='param.yaml'**

**extractor= FEE.RadiomicsFeatureExtractor(para_path)**

**result= extractor.execute(ori_path, lab_path)**

**T2WI_value = ((-0.0461) * (result['original_glszm_SmallAreaHighGrayLevelEmphasis']-858.855129370635)/523.622693874222**

**+(0.0721) * (result['log-sigma-3-0-mm-3D_firstorder_Mean']+7.10987898428323)/9.86574336008581**

**+(0.0143) * (result['wavelet-LHL_firstorder_Median']+0.497480301566474)/6.70861813994384**

**+(0.0026) * (result['wavelet-HLL_firstorder_Median']+0.50384535450867)/6.64617085861184**

**+(-0.0288) * (result['wavelet-HLL_firstorder_Skewness']+0.433378035999999)/0.857937526164875**

**+(-0.0650) * (result['wavelet-HLL_glcm_Correlation']-0.0799560479942196)/0.0842701277455332**

**+(-0.0293) * (result['wavelet-LLL_firstorder_10Percentile']-212.791719080578)/113.352771138532)**

**return T2WI_value**

**def T2flair():**

**ori_path=entry3.get()**

**lab_path=entry5.get()**

**para_path='param.yaml'**

**extractor= FEE.RadiomicsFeatureExtractor(para_path)**

**result= extractor.execute(ori_path, lab_path)**

**T2Flair_value = ((-0.0019) * (result['original_glrlm_LongRunHighGrayLevelEmphasis']-2240.14702016994)/1113.75171862019**

**+(-0.0192) * (result['original_glszm_HighGrayLevelZoneEmphasis']-1662.55764616878)/801.546612203378**

**+(-0.0517) * (result['log-sigma-3-0-mm-3D_glcm_ClusterShade']-99.099517136763)/457.10325677393**

**+(0.0181) * (result['wavelet-LLH_firstorder_Median']+6.49869639713872)/6.81144741424438**

**+(-0.0179) * (result['wavelet-HHH_firstorder_Mean']+0.00796741110982659)/0.225955014528202)**

**return T2Flair_value**

**def ADC():**

**ori_path=entry4.get()**

**lab_path=entry5.get()**

**para_path='param.yaml'**

**extractor= FEE.RadiomicsFeatureExtractor(para_path)**

**result= extractor.execute(ori_path, lab_path)**

**ADC_value = ((-0.0437) * (result['wavelet-LHL_firstorder_Skewness']+0.660421803092485)/1.02093354549596**

**+(-0.0140) * (result['wavelet-HLL_firstorder_Skewness']+0.740114255641618)/0.84046517671544**

**+(-0.0983) * (result['wavelet-HLH_firstorder_Median']+0.538403181971098)/8.43516762863539**

**+(0.0355) * (result['wavelet-HLH_firstorder_Skewness']-0.207093367872832)/0.643958301754604)**

**return ADC_value**

**def Rad_calculation():**

**Rad_result = T1c()+T2()+T2flair()+ADC()**

**lable6['text']=Rad_result**

**window = tk.Tk()**

**window.title("Radiomics signature calculation")**

**window.geometry("800x350")**

**tk.Label(window,text="Path of T1C",font=("Arial,12"),width=20,height=2).place(x=0,y=0)**

**tk.Label(window,text="Path of T2WI",font=("Arial,12"),width=20,height=2).place(x=0,y=40)**

**tk.Label(window,text="Path of T2Flair",font=("Arial,12"),width=20,height=2).place(x=0,y=80)**

**tk.Label(window,text="Path of ADC",font=("Arial,12"),width=20,height=2).place(x=0,y=120)**

**tk.Label(window,text="Segmentation",font=("Arial,12"),width=20,height=2).place(x=0,y=160)**

**entry1 = tk.Entry(window,show=None)**

**entry2 = tk.Entry(window,show=None)**

**entry3 = tk.Entry(window,show=None)**

**entry4 = tk.Entry(window,show=None)**

**entry5 = tk.Entry(window,show=None)**

**entry1.place(x=200,y=10,width=500,height=25)**

**entry2.place(x=200,y=50,width=500,height=25)**

**entry3.place(x=200,y=90,width=500,height=25)**

**entry4.place(x=200,y=130,width=500,height=25)**

**entry5.place(x=200,y=170,width=500,height=25)**

**tk.Button(window,text="Calculation",width=15,height=2,command=Rad_calculation).place(x=300,y=220)**

**tk.Label(window,text="Radiomics signature",font=("Arial,12"),width=20,height=2).place(x=0,y=280)**

**lable6 = tk.Label(window,font=("Arial,12"),width=20,height=2)**

**lable6.place(x=200,y=280)**

**window.mainloop()**

**Data Supplement S4: the Graphical User Interface (GUI) after running the python script**


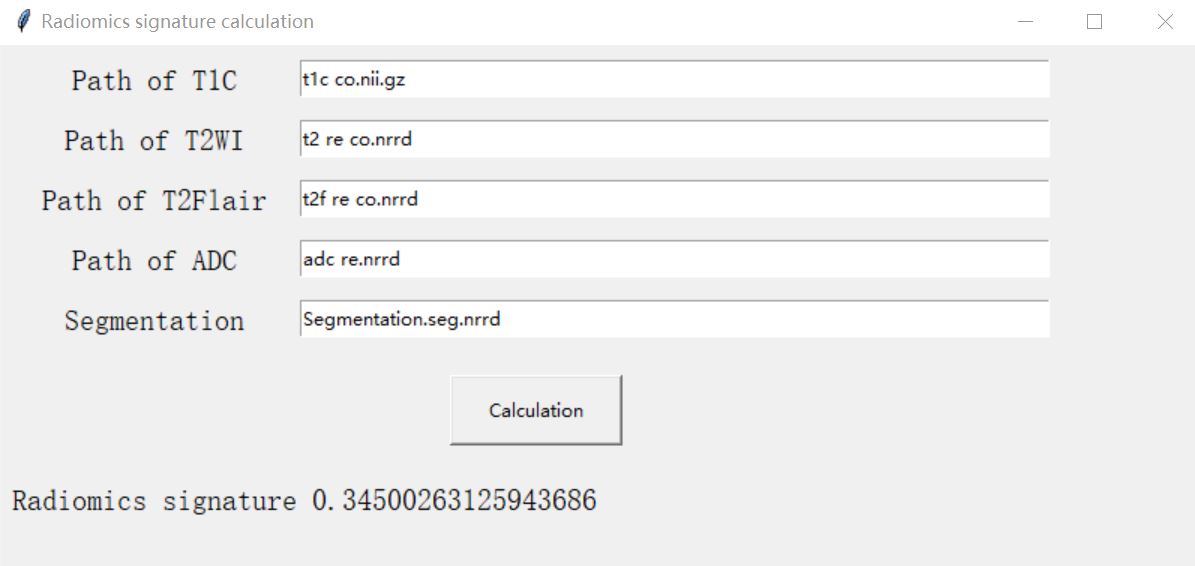


Users need put the python script, MRI sequences and the segmentation file in the same folder. After running the python script, users need input the file name in the corresponding textbox and click the Calculation button. Then, the Radiomics signature would be output below.
